# Noise-Aware Constitutive Compatibility Atlas for Hyperelastic Material Characterization

**DOI:** 10.64898/2026.10.12.758616

**Published:** 2026-10-14

**Authors:** Amirreza Asadi, Kaveh Laksari

## Abstract

Hyperelastic constitutive characterization is usually posed as selecting the best-fitting model, although several families can reproduce measured loading paths while predicting different responses elsewhere. We developed a noise-aware constitutive-response atlas to determine which families are compatible with an observed response over a prescribed deformation range and within a stated error tolerance. Each family forms a continuous response manifold in a common bounded deformation space, with exact nesting preserved. Standard uniaxial, biaxial, and shear tests are restricted projections of the complete response space, allowing the atlas to identify deformation states that resolve ambiguities left by conventional calibration. The output can contain several families, one family, or none, without forcing a single winner. We constructed the atlas for seven incompressible isotropic hyperelastic families using literature-based parameter ranges. Numerical certification and three controlled tests examined generating-family recovery, separation of responses distinct at the prescribed tolerance, and rejection of a withheld constitutive law. External validation used Treloar rubber and human-brain datasets. For Treloar rubber, uniaxial calibration retained the Mooney-Rivlin and one-term Ogden families; an atlas-selected equibiaxial measurement favored Mooney-Rivlin. For cortex, compression retained the one-term Ogden and second-order Yeoh families; withheld tension and shear favored one-term Ogden. The atlas thus preserves genuine ambiguity, exposes an inadequate candidate library, and identifies when another measurement is needed.

